# Association between Dietary Inflammatory Index (DII®) and depression and anxiety in the Mashhad Stroke and Heart Atherosclerotic Disorder (MASHAD) Study population

**DOI:** 10.1186/s12888-020-02663-4

**Published:** 2020-06-05

**Authors:** Hamideh Ghazizadeh, Mahdiyeh Yaghooti-Khorasani, Zahra Asadi, Reza Zare-Feyzabadi, Fatemeh Saeidi, Niloofar Shabani, Mahshid Safari-Ghalezou, Mehran Yadegari, Abolfazl Nosrati-Tirkani, Nitin Shivappa, James R. Hébert, Mohsen Moohebati, Gordon A. Ferns, Habibollah Esmaily, Majid Ghayour-Mobarhan

**Affiliations:** 1 Student Research Committee, School of Medicine, Mashhad University of Medical Sciences, Mashhad, Iran; 2 Metabolic Syndrome Research Center, School of Medicine, Mashhad University of Medical Sciences, Mashhad, Iran; 3 International UNESCO Center for Health-Related Basic Sciences and Human Nutrition, Mashhad University of Medical Sciences, Mashhad, Iran; 4 Social Determinants of Health Research Center, Mashhad University of Medical Sciences, Mashhad, Iran; 5 Department of Nutrition, faculty of Medicine, Mashhad University of Medical Sciences, Mashhad, Iran; 6 Cancer Prevention and Control Program, University of South Carolina, Columbia, SC 29208 USA; 7 Department of Epidemiology and Biostatistics, Arnold School of Public Health, University of South Carolina, Columbia, SC 29208 USA; 8 Connecting Health Innovations LLC, Columbia, SC 29250 USA; 9 Cardiovascular Research Center, School of Medicine, Mashhad University of Medical Sciences, Mashhad, Iran; 10 Division of Medical Education, Brighton & Sussex Medical School, Falmer, Brighton, Sussex UK

**Keywords:** Depression, Anxiety, Dietary inflammatory index, Diet

## Abstract

**Background:**

Systemic inflammation is emerging as an important factor in the etiology of psychiatric disorders such as depression and anxiety. Therefore, the inflammatory potential of the diet may also be an etiological factor for these conditions, and this may be estimated by calculating the dietary inflammatory index (DII**®**) score. We aimed to investigate the association between DII score and incidence of depression and anxiety among a representative sample in northeastern Iran.

**Methods:**

This cross-sectional study undertook in a sub-sample of 7083 adults aged 35 to 65 years recruited as part of Mashhad stroke and heart atherosclerotic disorder (MASHAD) cohort study population, and after excluding subjects with incomplete data. All participants completed the Beck Anxiety Inventory (BAI), the Beck Depression Inventory II (BDI-II), and a validated 65-item food frequency questionnaire (FFQ). Logistic regression was used to evaluate the association between DII score and depression/anxiety score.

**Results:**

Of the study participants, 37.1% (*n* = 2631) were found to have mild to severe depression, and 50.5% (*n* = 3580) were affected by mild to severe anxiety. After adjusting for confounding factors, in women, the third (OR: 1.41, 95% CI: 1.06–1.88, *p*-values< 0.05) and fourth quartiles (OR: 1.37, 95% CI: 1.03–1.83, *p*-values< 0.05) of DII score were associated with increased risk of a high depression score compared to the first quartile of DII score.

**Conclusion:**

There was a significant association between DII score and severe depression among women but not men in this Iranian population. In order to confirm the association between DII food score, depression, and anxiety, further research is required in different populations, and perhaps an intervention study.

## Background

Psychiatric disorders include a wide range of conditions; including major depressive disorder (MDD) and anxiety disorders [1, 2], that are associated with disability and morbidity [1, 3–5]. Anxiety and depression result from dysfunctions of the amygdala and hippocampus, the most important areas of the brain involved in the regulation of emotion [1, 2]. It has been estimated that at least 12% of adults have suffered or will suffer from an episode of depression in their lifetime and it is expected that depression will become the second most prevalent disease by 2020 [6, 7]. In addition, the prevalence of anxiety disorders is 7.3% in adults, globally [8]. The prevalence of anxiety in pre-adolescent children has been reported to be 2.6–41.2% [9]; also, the prevalence of depression in this group was reported to be 9% [10]. The prevalence of psychiatric disorders, major depressive disorder, and anxiety disorders in the Iranian population ≥ 18 years of age are 10.8, 3.0 and 8.4%, respectively [11].

Some etiologic factors for depression have been established; these include environmental and emotional instability and decreased levels of the neurotransmitters serotonin and norepinephrine [12]. Antidepressant medications modulate neurotransmitters and hormones receptor sensitivity; a common mechanism of antidepressants is reduction of β-adrenergic and enhancement of serotonergic and α-adrenergic sensitivity [13]. Antidepressants based on this mechanism are used to treat anxiety disorders and depression [13, 14]. Recently, it has been suggested that antidepressant therapy might influence serum concentrations of inflammatory markers; however, findings have been inconsistent [15–17]. A novel hypothesis has emerged suggesting that inflammation is an etiological factor for depression and anxiety. It has been proposed that pro-inflammatory cytokines could increase the risk of depression and anxiety, while anti-inflammatory or immunoregulatory cytokines may reduce this risk [14–16].

Pro-inflammatory markers include: tumor necrosis factor-α (TNF-α), C-reactive protein (CRP), interleukin-6 (IL-6), fibrinogen and interferon-gamma (IFN-γ), while interleukin-10 (IL-10) and IL-4 are anti-inflammatory [17, 18]. Increased systemic inflammation is associated with an unhealthy diet, obesity, hyperlipoproteinemia, alcohol use, hypertension, diabetes mellitus, physical inactivity, lower educational attainment, advancing age and genetic predisposition [19].

A Western dietary pattern can increase levels of inflammatory markers in the blood, while a Mediterranean dietary pattern can decrease these levels [20]. Previous studies have shown that adherence to a Western dietary pattern could increase the risk of depression, while Mediterranean dietary pattern plays a protective role against depression; although, some other studies have produced inconsistent results [7, 21–23].

The dietary inflammatory index (DII®) has been designed to quantify the potential inflammatory properties of a diet [24]. The relationships between DII and metabolic syndrome, mortality, cancer and cardiovascular diseases (CVDs) have been studied in several countries [20, 25–30]. Interest in the relationship between DII score with depression and anxiety worldwide has resulted in three systematic reviews/ meta-analyses [7, 31–33]. Our aim in this study was to quantify the possible inflammatory effects of diet on the occurrence of depression and anxiety in Mashhad, a city in northeastern Iran.

## Methods

### Study population

We used data from the Mashhad stroke and heart atherosclerotic disorder (MASHAD) study, a cohort study designed with the purpose of evaluating different cardiovascular disease (CVD) risk factors among 9704 residents of Mashhad city between 35 and 65 years of age who were free of chronic diseases at baseline [34]. In the current analysis, we excluded those participants who had incomplete data regarding dietary intake or depression or anxiety scores (*n* = 2621). Final analysis was undertaken in 7083 subjects. All participants provided informed written consent, and the study was approved by the Human Research Ethics Committee of Mashhad University of Medical Sciences (MUMS).

### Diagnosis of depression and anxiety

All participants completed depression and anxiety questionnaires which were collected by health care professionals and a nurse interviewer at baseline. These tests had previously been validated in the Iranian population [35, 36]. Psychometric tests were performed using the Beck Anxiety Inventory (BAI) to calculate an anxiety score that is explained as follows: a 0–7, minimal level of anxiety; 8–15, mild anxiety; 16–25, moderate anxiety and 26–63, severe anxiety. The Beck Depression Inventory II (BDI-II) was used to evaluate depression. The cut-offs used were as follows: 0–13, no, or minimal depression; 14–19, mild depression; 20–28, moderate depression; and 29–63, severe depression [37, 38].

### Dietary intake assessment

We utilized a semi-quantitative food frequency questionnaire (FFQ) containing 65 food items which has been validated previously. Each food parameter was questioned in 5 frequency groups and portion size [39]. The FFQ was completed by nutritionists at baseline. Diet Plan 6 software (Forestfield Software Ltd., Horsham, West Sussex and UK) was used for analyzing macro/ and micronutrients intake.

### Dietary inflammatory index (DII®)

The DII score was calculated to determine the inflammatory possible effect of the diet. The concept and computation of the DII score have been previously described by Asadi et al. [40]. Computation of the DII score is based on the reported consumption of up to 45 food parameters and in this study, a total of 28 of 45 food parameters derived from the FFQ were used (these 28 food parameters have been defined before by us in Asadi et al. [40] study). DII is ranged from − 8.87 (the most anti-inflammatory diet) to + 7.98 (the most pro-inflammatory diet) [41]. All micronutrients and macronutrients were adjusted for total energy intake. We categorized the DII score into quartiles, defined as Q1, Q2, Q3 and Q4 (Q1 being the most anti-inflammatory quartile and Q4 the most pro-inflammatory quartile; Q1 was used as the reference group for comparisons).

### Assessment of other variables

Demographic and socioeconomic characteristics, medical history, laboratory tests, systolic and diastolic blood pressure (SBP and DBP), anthropometric criteria, physical activity level (PAL) and laboratory tests for all participants were collected by health care professionals and a nurse interview. Method of these assessments were introduced previously by Asadi et al. [40].

### Statistical analysis

Statistical analysis was performed using the SPSS® version 20 (SPSS, Chicago, IL). The Kolmogorov- Smirnov test was used to check the normality of data. The chi-square and Student t-tests were used to compare qualitative and quantitative normal data, respectively. Several variables including TG and hs-CRP were found to be non-normal, even after logarithmical transformation; therefore, the Kruskal-Wallis test and post-hoc pairwise comparisons were performed in order to compare these variables across quartiles of depression/anxiety score. Normally distributed data were presented as mean ± standard deviation (SD), while median and interquartile range (IQR) were reported for non-normally distributed data. A logistic regression analysis was used to assess the relationship between DII score and depression/anxiety level, and the odds ratios (OR) and 95% confidence intervals (95%CI) were calculated. Models were then adjusted for possible confounding factors including age (35–44, 45–54, 55–65 years), BMI, smoking status (never, ex-smoking, current smoking), education level (low, moderate, high), marital status (single/divorced/widow and married) and PAL, serum hs-CRP and dyslipidemia and these adjusted ORs and 95% CIs were reported.

## Results

Final analysis was undertaken in a sample of 7083 participants of the MASHAD study after excluding persons who did not complete the FFQ, or had missing data on the depression or anxiety score. We divided the sample population based on the depression severity score according to the BDI-II into four groups (number of person with no or minimal depression = 4452, mild depression = 1251, moderate depression = 924, and severe depression = 456). We also divided this population based on the anxiety severity score according to the BAI into four groups (number of persons with no or minimal anxiety = 3503, mild anxiety = 1904, moderate anxiety = 1026, and severe anxiety = 650).

In Table 1, we show the comparison of the four groups of depression and anxiety severity score in the study population. The percentage of women increased with the severity of depression and anxiety (*p*-value < 0.001). Participants in the highest quartile of depression and anxiety score tended to be less educated (*p*-value < 0.001). Increasing depression and anxiety scores were associated with an increasing percentage of smokers (either ex-smoker or current smoker) (*p*-value < 0.001). Participants with the highest depression and anxiety score had higher levels of PAL (*p*-values =0.001), were shorter (*p*-value < 0.001), had higher body mass index (BMI) (*p*-value < 0.001), and hip circumference (HC) (*p*-values < 0.05). Dyslipidemia and higher levels of high sensitivity C-reactive protein (hs-CRP) were associated with higher depression and anxiety severity score, while fasting blood glucose (FBG), hypertension, and diabetes mellitus prevalence were higher only among those participants in the highest quartile of anxiety score compared to the lowest (*p*-value < 0.05). The percentage of participants who were married fell with increasing the severity of depression and anxiety scores (*p*-value < 0.001).

**Table 1:** Demographic and biochemical characteristics of individuals in groups of Depression and Anxiety

	Depression severity score					Anxiety severity score				
	No or minimal *N*=4452	Mild *N*=1251	Moderate *N*=924	Severe *N*=456	Sig.	No or minimal *N*=3503	Mild *N*=1904	Moderate *N*=1026	Severe *N*=650	Sig.
**Sex (female), % (N)**	52.60 (2342)	62.60 (783)	65.90 (608)	74.60 (340)	<0.001	48.70 (1795)	61.90 (1178)	69.10 (709)	74 (481)	<0.001
**Age (y)**	48.91 ± 7.98	49.12 ± 7.84	48.90 ± 7.99	49.08 ± 7.62	0.832	49.10 ± 7.96	48.76 ± 7.99	49.11 ± 7.74	48.53 ± 7.90	0.209
**Education; % (N)**										
**Low (trade school)**	53.80 (2395)	51.20 (640)	54.50 (504)	60 (274)	0.013	52.20 (1831)	52.80 (1006)	57 (585)	60.50 (393)	<0.001
**Moderate (high school)**	33 (1469)	36.20 (453)	36.60 (338)	35.40 (161)	0.049	33.80 (11.82)	35.80 (682)	33.10 (339)	33.30 (217)	0.339
**High (university)**	13.20 (588)	12.60 (158)	8.90 (82)	4.60 (21)	<0.001	14 (490)	11.40 (216)	10 (102)	6.20 (40)	<0.001
**Smoking status; % (N)**										
**Non smoker**	71 (3160)	66.90 (838)	62.90 (581)	56.80 (259)	<0.001	71.30 (2499)	67.60 (1288)	64.70 (664)	59.20 (385)	<0.001
**Ex – smoker**	10 (445)	10.20 (128)	9.30 (86)	11 (50)	0.791	9.80 (344)	9.90 (188)	10.20 (105)	11.10 (72)	0.790
**Current smoker**	19 (847)	22.90 (286)	27.80 (257)	32.20 (147)	<0.001	18.80 (660)	22.50 (428)	25 (257)	29.70 (193)	<0.001
**Marital status; % (N)**										
**Married**	94 (4186)	93 (1162)	89.90 (831)	87.90 (401)	0.676	94.40 (3304)	92.40 (1759)	91.40 (938)	89.10 (579)	0.514
**Single/ divorced/ widow**	6 (266)	7 (89)	10.10 (93)	92.10 (55)	<0.001	5.60 (199)	7.60 (145)	8.60 (88)	10.90 (71)	<0.001
**PAL**	1.56 ± 0.27 ^a^	1.57 ± 0.27 ^b^	1.59 ± 0.28 ^c^	1.61 ± 0.28 ^ab^	0.001	1.56 ± 0.28 ^a b c^	1.58 ± 0.27 ^a^	1.59 ± 0.26 ^b^	1.61 ± 0.28 ^c^	<0.001
**Weight (kg)**	72.08 ± 12.83	72.33 ± 12.60	71.76 ± 12.74	71.82 ± 13.46	0.745	72.14 ± 12.98	71.85 ± 12.55	72.06 ± 12.75	72.34 ± 12.83	0.812
**Height (cm)**	161.65 ± 0.09 ^a b c^	160.11 ± 0.09 ^ad^	159.64 ± 0.09 ^be^	158.02 ± 0.09 ^c d e^	<0.001	162.15 ± 0.09 ^a b c^	160.30 ± 0.09 ^a d e^	159.12 ± 0.09 ^b d^	158.51 ± 0.08 ^c e^	<0.001
**BMI (kg/ m²)**	27.61 ± 4.54 ^a b c^	28.25 ± 4.63 ^a^	28.22 ± 4.88 ^b^	28.83 ± 5.25 ^c^	<0.001	27.45 ± 4.50 ^a b c^	28.01 ± 4.64 ^a d e^	28.53 ± 4.94 ^b d^	28.84 ± 4.90 ^c e^	<0.001
**WC (cm)**	95.43 ± 11.68	95.89 ± 12.18	95.13 ± 12.31	95.75 ± 13.12	0.464	95.34 ± 11.68	95.22 ± 11.95	95.95 ± 12.26	96.35 ± 12.82	0.091
**HC (cm)**	103.25 ± 8.76	103.97 ± 9.18	103.91 ± 9.51	104.09 ± 9.95	0.013	103.03 ± 8.70 ^a b^	103.49 ± 8.96 c	104.27 ± 9.42 ^a^	105.03 ± 9.98 ^b c^	<0.001
**WHR**	0.92 ± 0.07 ^a^	0.92 ± 0.07	0.91 ± 0.08 ^a^	0.92 ± 0.08	0.013	0.92 ± 0.07	0.92 ± 0.07	0.92 ± 0.08	0.92 ± 0.08	0.011
**Diabetes; % (N)**	8.76 (390)	9.67 (121)	8.76 (81)	10.74 (49)	0.439	8.40 (3503)	9.93 (189)	7.90 (81)	11.85 (77)	0.009
**Hypertension; % (N)**	25.90 (1152)	24.10 (301)	24 (222)	21.05 (96)	0.086	26.21 (918)	22.79 (434)	24.70 (253)	25.54 (166)	0.048
**Dyslipidemia; % (N)**	85.60 (3810)	88.33 (1105)	89.70 (829)	92.54 (422)	<0.001	85.13 (2982)	88.13 (1678)	90.35 (927)	89.08 (579)	<0.001
**FBG (mg/dl)**	91.60 ± 37.24	92.35 ± 39.71	92.51 ± 41.99	94.36 ± 42.88	0.488	91.06 ± 36.90 ^a^	92.92 ± 38.48	91.02 ± 39.22 ^b^	96.20 ± 46.98 ^a b^	0.010
**Cholesterol (mg/dl)**	191.28 ± 39.30	192.42 ± 38.79	191.10 ± 38.08	191.26 ± 37.48	0.815	190.75± 39.09	192.38 ± 8.19	193.05 ± 40.09	190.05 ± 38.34	0.189
**TG (mg/dl)**	122 (85, 175)	125 (86, 180)	124 (89.25, 176)	119 (89.75, 172)	0.214	121 (85, 173)	124 (87, 179)	124 (89, 179)	122.50 (87, 174.25)	0.144
**LDL-C(mg/dl)**	118.92 ± 34.66	119.08 ± 34.27	117.71 ± 35.22	118.34 ± 34.86	0.775	118.66 ± 34.32	119.13 ± 33.98	119.49 ± 37.11	117.02 ± 34.67	0.509
**HDL-C (mg/dl)**	41.93 ± 9.54	41.80 ± 9.49	41.43 ± 9.16	41.26 ± 9.29	0.294	41.87 ± 9.58	41.74 ± 9.43	41.71 ± 9.48	41.70 ± 9.93	0.937
**Serum hs-CRP (mg/L)**	1.43 (0.91, 2.92) ^a b^	1.65 (0.94, .21)	1.60 (0.94, 3.71) ^a^	1.71 (1, 3.85) ^b^	<0.001	1.40 (0.90, 2.80) ^a b c^	1.59 (0.95, 3.35) ^a^	1.58 (0.96, 3.39) ^b^	1.74 (1.01, 3.94) ^c^	<0.001

*Abbreviations*: *PAL* physical activity level, *BMI* body mass index, *WC* waist circumference, *HC* hip circumference, *WHR* waist – to- hip ratio, *FBG* fasting blood glucose, *TG* triglyceride, *LDL-C* low density lipoprotein cholesterol, *HDL-C* high density lipoprotein cholesterol, *hs-CRP* high sensitivity C-reactive protein

Data are presented as mean ± standard deviation and median (interquartile range) for continuous variables and as percentage (number) for categorical variables. Independent sample t-test, Kruskal-Wallis test, and post-hoc pairwise comparisons are used where appropriate

Two groups in a row with similar alphabets had a significant difference (*P* < 0.05)

In Table 2, we show the DII score between males and females in quartiles for depression and anxiety severity score; no significant differences were found.

**Table 2:** Dietary inflammatory index (DII) in groups of Depression and Anxiety

		Depression severity score					Anxiety severity score				
		No or minimal *N* = 4452	Mild *N* = 1251	Moderate *N* = 924	Severe *N* = 456	***p***-value	No or minimal *N* = 3503	Mild *N* = 1904	Moderate *N* = 1026	Severe *N* = 650	***p***-value
**DII**	**Males**	0.57 (−0.37, 1.38)	0.53 (−0.71, 1.45)	0.61 (− 0.56, 1.36)	0.44 (− 0.41, 1.31)	0.557	0.60 (− 0.41, 1.42)	0.51 (− 0.45, 1.29)	0.55 (− 0.39, 1.40)	0.43 (− 0.77, 1.52)	0.402
	**Females**	0.39 (− 0.75, 1.41)	0.30 (− 0.81, 1.34)	0.38 (− 0.77, 1.38)	0.31 (− 0.86, 1.22)	0.361	0.41 (− 0.77, 1.40)	0.34 (− 0.77, 1.34)	0.35 (− 0.73, 1.43)	0.22 (− 0.97, 1.23)	0.082

Values are expressed as median and inter-quartile range. Between-group comparisons were assessed by Kruskal–Wallis test

In Table 3, we show the scores for depression and anxiety by quartiles of DII score in which the minimal depression/anxiety level is the reference group. After adjusting for confounding factors (for age [35–44, 45.54 and 55–65], BMI, smoking status, education level, marital status, PAL, hs-CRP and dyslipidemia), we found that the odds of severe depression was higher in women in the third (OR: 1.41, 95% CI: 1.06–1.88, *p*-values = 0.019) and fourth quartiles (OR: 1.37, 95% CI: 1.03–1.83, *p*-values = 0.032) for DII compared to the first quartile. However, other categories of DII did not show a significant association with depression. In addition, we found no relationship between DII score and anxiety in either males or females.

**Table 3:** Adjusted odds ratios of having severe depression or anxiety symptoms associated with DII among men and women

Gender	DII (Q1 = reference group)	**Depression severity risk** (minimal depression quartile as the reference group)								
		Reference group and mildly affected group			Reference group and moderately affected group			Reference group and severely affected group		
		**OR**	**95% CI**	**Sig.**	**OR**	**95% CI**	**Sig.**	**OR**	**95% CI**	**Sig.**
Male	Q2	0.74	0.55–1	0.051	0.81	0.57–1.16	0.254	1.27	0.74–2.15	0.381
	Q3	0.87	0.66–1.15	0.335	1.14	0.82–1.57	0.441	0.97	0.56–1.68	0.922
	Q4	1.15	0.87–1.53	0.323	1.22	0.86–1.72	0.260	1.11	0.63–1.96	0.723
Female	Q2	0.92	0.77–1.10	0.369	0.91	0.74–1.11	0.356	1.23	0.92–1.64	0.171
	Q3	1.02	0.85–1.22	0.849	1.07	0.87–1.31	0.534	1.41	1.06–1.88	**0.019**
	Q4	1.16	0.98–1.39	0.089	1.12	0.92–1.37	0.257	1.37	1.03–1.83	**0.032**
Gender	DII (Q1 = reference group)	**Anxiety severity risk** (minimal anxiety quartile as the reference group)								
		Reference group and mildly affected group			Reference group and moderately affected group			Reference group and severely affected group		
		**OR**	**95% CI**	**Sig.**	**OR**	**95% CI**	**Sig.**	**OR**	**95% CI**	**Sig.**
Male	Q2	1.24	0.97–1.58	0.091	0.91	0.64–1.29	0.601	0.88	0.56–1.38	0.585
	Q3	1.20	0.94–1.53	0.144	1.15	0.83–1.59	0.400	0.67	0.42–1.07	0.093
	Q 4	1.22	0.94–1.59	0.127	1.09	0.76–1.55	0.647	1.21	0.78–1.87	0.400
Female	Q2	1.06	0.86–1.32	0.579	1.04	0.81–1.33	0.772	1.20	0.89–1.62	0.234
	Q3	1.06	0.85–1.32	0.603	0.96	0.74–1.24	0.731	1.07	0.78–1.47	0.657
	Q4	1.08	0.88–1.33	0.471	0.95	0.74–1.22	0.705	1.33	1–1.78	0.052

*Abbreviations*: *Q* quartile, *OR* odds ratio, *CI* confidence interval

ORs were adjusted for age (35–44, 45.54 and 55–65), body mass index, smoking status, education level, marital status, physical activity level, high sensitivity C-reactive protein, and dyslipidemia

## Discussion

The MASHAD cohort study subgroup consisted of 7083 men and women in the age range of 35 to 65 years. Our aim was to assess the association between DII, depression and anxiety scores in the population of Mashhad city, in Iran. In this population, 37.1% (*n* = 2631) of participants had mild to severe depression and 50.6% (*n* = 3580) suffered from mild to severe anxiety. We found that women in the third and fourth quartiles of DII score had a higher odd of moderate and severe depression levels, respectively compared to those in the first quartile. However, it should be noted that there was not a dose-dependent effect and the relationship between DII and severe depression was weaker than the relationship between DII and moderate depression, and this was as a potentially an artifice of chance.

Exposure to stressful psychosocial situations activates sympathetic nervous system and hypothalamic–pituitary–adrenal (HPA) axis; that both of them have immunomodulatory functions [42, 43]. Thus, in psychiatric diseases such as depression, anxiety disorders, and schizophrenia inflammatory markers and cytokines are increased [43–45]. In addition, several cases of major depressive disorders and anxiety disorders do not response to conventional antidepressants, in patients with a serum CRP level > 3 mg/L [46]. Also, Firth et al. recently showed in a review article that inflammation induced by pro-inflammatory nutrients in the diet can impair cognitive function [47]. They also demonstrated that a novel treatment of depression is based on reducing inflammation and specially by altering the diet of depressed people from pro-inflammatory to anti-inflammatory diet [47]; though, changing in the diet did not affect anxiety score [47, 48]. Therefore, pro-inflammatory diets may enhance levels of inflammatory cytokines that could affect the depression and anxiety disorders.

In our study, there was a positive association between higher DII score and the severity of depression in women. Also, the percentage of women with mild to severe depression (65.79%) and anxiety (66.14%) was higher compared to men. Several studies have demonstrated that the incidence and prevalence of psychiatric disorders, such as depression and anxiety, are greater in women [49]. The immune system in women is stronger than men because estrogen enhances humoral immunity; thus, autoimmune diseases are more prevalent in females [50]. Therefore, immune markers and inflammatory markers tend to be higher in females [50]. In addition, the consumption of pro-inflammatory nutrients increases the level of inflammatory markers [18].

Sanaeei et al. demonstrated that a high alternative healthy eating index (AHEI) was associated with lower anxiety and depression score among Iranian population [51]. Gibson-Smith et al. showed that participants with depression/anxiety disorders consumed a poorer diet (with lower alternative healthy eating index [AHEI] and Mediterranean Diet Score [MDS]) in a 9-years follow-up cohort study [52]. HEI and AHEI reflect the diet quality and are associated with lower DII [53–55]. Philips et al. showed a positive association between anxiety and DII (OR 1.60, 95% CI 1.15–2.24, *p* = 0.006) by comparing the highest and the lowest tertiles of DII [56]. Our results were inconsistent with these studies. However, in accordance with our results, Firth et al. showed in a review article that diet has no effect on anxiety score [47]. It is notable that our study was a cross-sectional study while the studies referred to above, were cohort studies which did not find an increased risk of any psychiatric disease.

Several previous studies have reported associations between DII and depression/anxiety. However, the results of these studies have been inconsistent. Sánchez-Villegas et al. and Shivappa et al. showed a positive relationship between higher DII score and depression [7, 23]. Phillips et al. demonstrated positive relationships between both depressive symptoms and anxiety with DII [56]. Also, Bergmans et al. reported that higher DII score causes over two fold odds of depression; although, the association between DII and anxiety was not significant in their study [57]. Firth et al. demonstrated positive association between DII and depression [58].

Sánchez-Villegas et al. undertook a 10-year follow-up cohort study in 15,093 university graduates of Navarra University in Spain using an FFQ with 28 items [23]. They evaluated the effect of DII only on the incidence of depression, in the way of clinical diagnosis of depression, by a physician and found a positive association. In their population, they observed 1051 cases of depression after 10 years; i.e., only 7% of their sample size developed depression after follow-up duration. This result is very different to our results based on depression prevalence (37.14% had mild to severe depression score); this difference might be due to differences in the study population; both the sample size and the sample type. Their sample consisted of university graduates and our sample reflected a spectrum of education from trade school to university graduates. In our sample there was an inverse association between education level and severe depression. Based on their data and ours, it appears that more education is protective against depression. Also, lower education levels were associated with higher levels of anxiety in the present study that was not noticed by Sánchez-Villegas et al. Shivappa et al. followed-up 6438 Australian women with a mean age of 52 years for 12 years in a cohort study [7]. In their study, women with the lowest level of DII had an approximately 20% lower risk of developing depression compared with women with the highest DII scores. Phillips et al. have done a two-year cross-sectional study in a population of 2047 men and women in Ireland [56]. They employed an FFQ with 26 items and showed positive relation between DII score and both depression and anxiety. They showed that participants with the highest DII score had significantly increased level of depressive symptoms and anxiety in females; though, no association was found in males. In a cross-sectional study in the United States, Bergmans et al. assessed the association between DII, depression and anxiety in a population of 11,592 adults (older than 20 years old) in 2007–2012. There was no significant association between anxiety and DII; however, more than a two-fold odds of depression was observed between the highest versus the lowest quintile of DII. Sample size and age range of the population were different to our study. In addition, two other cross-sectional studies have been conducted in Iran, the city of Tehran, around the subject of DII and depression and anxiety with lower sample size, in young adults aged 18–35 years and female adolescents aged 15–18 years; in which their results were similar to ours [59, 60]. Futhermore, Firth et al. demonstrated in a population sample of 14,619 persons with major depressive disorder (MDD) and 54,010 persons in control group, that patients with MDD consumed significantly higher levels of total energy, sugar, carbohydrates, total and saturated fat and protein and the DII score was higher in MDD patients (B = 0.031, SE = 0.013, *p*-value = 0.014) compared to controls [58].

Another finding of our study was that as the severity of depression and anxiety increased, the percentage of smokers (ex-smoker and current smoker) also increased. This is in agreement with the results of Shivappa et al. who reported that women who were current smokers, were at higher risk of depression [7]. In addition, Paperwalla et al. reported that nicotine withdrawal may induce depression, because of the effects of nicotine on acetylcholine (one type of neurotransmitter) receptors in brain; however, current smoker participants had higher levels of depression/anxiety than ex-smoker participants [61]. Joseph et al. demonstrated the cause and effect relationship between smoking and depression, in which cigarette smoking and thereby nicotine dependence increases the risk of depressive symptoms [62].

A surprising finding of the current study was that higher PAL was directly related to the severity of both depression and anxiety. Our results were in contrast to the findings of previous studies about the association between physical activity and incidence of depressive symptoms and anxiety. Shivappa et al. showed that women who had higher risk of depression tended to have lower activity level [7]. Dunn et al. in a review article demonstrated that dose-response physical activity could reduce depressive and anxiety symptoms [63]. As well as, Weyerer et al. showed in German people, those who reported no physical activity were 3.15 times at higher risk for having moderate to severe depression [64]. Ströhle et al. and Dunn et al. suggested physical activity for preventing and treating depression and anxiety disorders (except phobias and Posttraumatic Stress Disorder [PTSD] that are linked to specific situations or cues) [63, 65]. These studies included exercises like aerobic exercise and resistance training for measuring the activity level; we categorized physical activity level as three categories, including at work, non-work and in bed [40]. Therefore, stress induced by occupational environment could affect our results; since Croon et al. and Urilch et al. showed that stressful jobs interact with the psychosocial status and wellbeing [66, 67]. In addition, this inverse finding might be related to the advice given to the group with severe depression and anxiety, to undertake more activity level (as exercise); because this subsample of the population had higher levels of cardiovascular disease risk factors such as diabetes, HTN, dyslipidemia and BMI.

Those participants who were shorter, had higher BMI, and HC were more likely to experience depression and anxiety. To explain this finding, we can refer to the influence of cardiovascular disease risk factors on activating inflammation process [19]. Ruiz-Canela et al. showed a positive relationship between DII and BMI, waist circumference, and obesity [27]. Therefore, if we accept the effect of inflammatory process on the incidence of depression and anxiety, we could resolve the association between higher BMI and HC with the occurrence of depressive symptoms and anxiety disorders.

The prevalence of diabetes mellitus and the level of FBG were associated with the severity of anxiety; though, there was no association between them and the severity of depression. Previous studies have reported results that conflict to this finding; i.e., Goldney et al. reported that the prevalence of depression in diabetics is 7% greater than its prevalence in non-diabetics [68]. Mezuk et al. showed in a meta-analysis between 1950 and 2007, that depression is associated with a 60% increased risk of type 2 diabetes, though type 2 diabetes is associated with only modest increased risk of depression [69]. Furthermore, a systematic review found positive associations between diabetes and both anxiety disorders and elevated anxiety symptoms which are in agreement with our findings [70]. A hypothesis is suggested that in diabetic patients, hyperglycemia is exacerbated experimented during psychiatric situations [71]. For describing this hypothesis we can refer to the role of hypothalamic pituitary adrenocortical (HPA) system and its modulation by corticotrophin-releasing hormone (CRH); in major depression the function of glucocorticoid receptor is impaired which cause an excessive release of neurohormones like CRH and also corticosteroids; also, in anxiety disorders such as panic attack, CRH and corticosteroids levels are elevated [72]. Corticosteroids elevate blood sugar through gluconeogenesis; therefore, this may help to explain the positive associations between psychiatric disorders and diabetes.

Hypertension was associated with the severity of anxiety, though it was not associated with depression in this study; the possible mechanism is the activation of sympathetic system in psychosocial stress; thereby, elevated blood pressure [43]. Results from other studies are variable. Grimsrud et al. concluded in a cohort study in sample of South African adults, that hypertension in the absence of other chronic physical conditions was not associated with mental health outcomes, while there was an association in the presence of other chronic physical conditions [73]. Also, Jonas et al. considered anxiety and depression as predictors for the later incidence of hypertension [74].

In the current study, higher levels of serum hs-CRP were associated with the severity of both depression and anxiety. This finding is in agreement with this theory about the association of chronic low-grade inflammation with depression and anxiety [75]. Köhler-Forsberg et al. indicated that higher CRP level is associated with the severity of depressive symptopms among females; though, no significant association was determined among males [75].

This study has several strengths. It was designed with a large sample size (*n* = 7083). We were able to adjust for many potential confounding factors. In contrast to some previous studies, we considered both depression and anxiety in our study. Some limitations of the current study must be acknowledged. First, as a cross-sectional study we do not know the temporal ordering of diet and anxiety or depression. Thus, it is more difficult to infer causality. Moreover, neurohormones like the levels of CRH, corticosteroids and mineralocorticoids, and also other inflammatory cytokines such as interleukins and erythrocyte sedimentation rate (ESR) (that is increases in inflammatory situations) were not considered in our evaluation. Additionally, the FFQ is known to be subject to reporting error (i.e., information bias) [76, 77].

Given the alarming rise in anxiety and depression globally, more investigations, preferably of cohort design should address this promising area of diet-associated inflammation. These studies should consider genetic and neurohormonal factors.

## Conclusion

We found a significant association between third and fourth quartiles of DII score and severe depression level in this cross-sectional study among women, in the city of Mashhad, in Iran; even though, no significant association was observed between the DII score and less severe depression. Additionally, there was no significant association between DII score and anxiety. Given that other studies have produced promising, but equivocal results, further investigations are needed for confirming these associations.

Disclosure: James R. Hébert owns controlling interest in Connecting Health Innovations LLC (CHI), a company that has licensed the right to his invention of the dietary inflammatory index (DII®) from the University of South Carolina in order to develop computer and smart phone applications for patient counseling and dietary intervention in clinical settings. Nitin Shivappa is an employee of CHI.

## Data Availability

Data sharing not applicable to this article as no datasets were generated or analyzed during the current study.

## Abbreviations

BAI: Beck Anxiety Inventory
BDI-II: Beck Depression Inventory II
CHI: Connecting Health Innovations LLC
DII: Dietary inflammatory index
ESR: Erythrocyte sedimentation rate
FFQ: Food frequency questionnaire
HPA: Hypothalamic pituitary adrenocortical
CRH: Corticotrophin-releasing hormone
Hs-CRP: High sensitivity C-reactive protein
BMI: Body mass index
HC: Hip circumference
FBG: Fasting blood glucose
PTSD: Phobias and Posttraumatic Stress Disorder
HTN: Hypertension
CVD: Cardiovascular disease
PAL: Physical activity
SD: Standard deviation
IQR: Interquartile range
MASHAD: Mashhad Stroke and Heart Atherosclerosis Disorder
HDL: High density lipoprotein
LDL: Low density lipoprotein
TG: Triglycerides
WC: Waist circumference
DBP/SBP: Diastolic/systolic blood pressure
FBG: Fasting blood glucose
OR: Odds ratio
Q: Quartile
